# Novel Genetic Variants of *ALG6* and *GALNTL4* of the Glycosylation Pathway Predict Cutaneous Melanoma-Specific Survival

**DOI:** 10.3390/cancers12020288

**Published:** 2020-01-24

**Authors:** Bingrong Zhou, Yu Chen Zhao, Hongliang Liu, Sheng Luo, Christopher I. Amos, Jeffrey E. Lee, Xin Li, Hongmei Nan, Qingyi Wei

**Affiliations:** 1 Department of Dermatology, The First Affiliated Hospital of Nanjing Medical University, Nanjing 210029, Jiangsu, China; zhoubingrong@njmu.edu.cn; 2 Duke Cancer Institute, Duke University Medical Center, Durham, NC 27710, USA; yu.chen.zhao@edu.uwaterloo.ca (Y.C.Z.); hongliang.liu@duke.edu (H.L.); 3 Department of Population Health Sciences, Duke University School of Medicine, Durham, NC 27710, USA; 4 Department of Biostatistics and Bioinformatics, Duke University School of Medicine, Durham, NC 27710, USA; sheng.luo@duke.edu; 5 Institute for Clinical and Translational Research, Baylor College of Medicine, Houston, TX 77030, USA; chris.amos@bcm.edu; 6 Department of Surgical Oncology, the University of Texas M. D. Anderson Cancer Center, Houston, TX 77030, USA; jelee@mdanderson.org; 7 Channing Division of Network Medicine, Department of Medicine, Brigham and Women’s Hospital, Boston, MA 02115, USA; xl16@iu.edu (X.L.); hnan@iu.edu (H.N.); 8 Department of Epidemiology, Fairbanks School of Public Health, Indiana University, Indianapolis, IN 46202, USA; 9 Department of Medicine, Duke University School of Medicine, Durham, NC 27710, USA

**Keywords:** cutaneous melanoma, expression quantitative trait loci, glycosylation, single-nucleotide polymorphism, survival analysis

## Abstract

Because aberrant glycosylation is known to play a role in the progression of melanoma, we hypothesize that genetic variants of glycosylation pathway genes are associated with the survival of cutaneous melanoma (CM) patients. To test this hypothesis, we used a Cox proportional hazards regression model in a single-locus analysis to evaluate associations between 34,096 genetic variants of 227 glycosylation pathway genes and CM disease-specific survival (CMSS) using genotyping data from two previously published genome-wide association studies. The discovery dataset included 858 CM patients with 95 deaths from The University of Texas MD Anderson Cancer Center, and the replication dataset included 409 CM patients with 48 deaths from Harvard University nurse/physician cohorts. In the multivariable Cox regression analysis, we found that two novel single-nucleotide polymorphisms (SNPs) (*ALG6* rs10889417 G>A and *GALNTL4* rs12270446 G>C) predicted CMSS, with an adjusted hazards ratios of 0.60 (95% confidence interval = 0.44–0.83 and *p* = 0.002) and 0.66 (0.52–0.84 and 0.004), respectively. Subsequent expression quantitative trait loci (eQTL) analysis revealed that *ALG6* rs10889417 was associated with mRNA expression levels in the cultured skin fibroblasts and whole blood cells and that *GALNTL4* rs12270446 was associated with mRNA expression levels in the skin tissues (all *p* < 0.05). Our findings suggest that, once validated by other large patient cohorts, these two novel SNPs in the glycosylation pathway genes may be useful prognostic biomarkers for CMSS, likely through modulating their gene expression.

## 1. Introduction

Cutaneous melanoma (CM) is one of the most lethal malignancies of the skin [1]. It is estimated that 96,480 new CM cases will be diagnosed in 2019 in the United States, accounting for about 5.5% of all new cancer cases, and 7230 patients will die of the disease in 2019 [2]. Age, sex, distant metastasis, ulceration, mitosis, and Breslow thickness are known to affect the prognosis of CM patients [3]. In addition, genetic variants in some genes of critical biological pathways may also play an important role in the prognosis [4].

Single-nucleotide polymorphisms (SNPs) are the common form of genetic variants that may affect gene expression and functions, likely leading to the development and progression of CM [5]. However, in genome-wide association studies (GWASs), few functional SNPs have been found to be associated with the prognosis of CM patients [6,7]. This is because a hypothesis-free GWAS always focuses on the most important SNP/genes with a strict *p* value after multiple tests correction for a large number of SNPs in some nascence comparisons. In the post-GWAS era, one can use available genotyping data from multiple previously published GWAS datasets in a hypothesis-driven approach to perform a biological pathway gene-set analysis with a narrow focus on the SNPs for more relevant comparisons. This approach gives investigators an improved statistical power to more likely identify novel functional loci with minor but detectable effects; therefore, investigators are able to further examine the functional relevance of these loci to unravel potential mechanisms underlying the observed associations [8].

Glycosylation refers to the formation of glycoside bonds between carbohydrates and amino acid residues in proteins catalyzed by glycosyltransferases, which eventually leads to changes in the protein products in cells [9]. As a post-translational modification, glycosylation plays an important role in the regulation of cellular protein functions during cell growth and differentiation, likely affecting the progression of tumor cells [10]. Thus far, hundreds of glycosyl groups have been identified as capable of binding to proteins or lipids and glycosylating to form glycoproteins, glycolipids, and glycans outside the cell membrane [11].

Depending upon a particular glycan, glycosylation is divided into N-linked glycosylation, O-linked glycosylation, C-mannosylation, glypiation, and phospho-glycosylation [12]. For example, the ALG6 alpha-1,3-glucosyltransferase *(ALG6*) gene, located on chromosome 1p31.3, encodes a protein that adds the first glucose residue to a lipid-linked oligosaccharide precursor, which is essential for N-linked glycosylation [13]. Another polypeptide N-acetylgalactosaminyltransferase 18 (*GALNTL4*) gene, also known as *GALNT18,* is located on chromosome 11p15.4, encoding a protein catalyzing the initial reaction in O-linked oligosaccharide biosynthesis [14]. In general, glycosylation of proteins can affect the spatial structure and stability of peptide chains and participate in cell signal transduction, recognition and adhesion, receptor activation, and other biological behaviors. Thus, aberrant glycosylation and modification may affect the proliferation, apoptosis, invasion, metastasis, drug resistance, and immune escape of tumor cells [15]. For example, using a systems biology approach to assess glycosylation in matched samples of primary and metastatic melanoma, one study found an increased core fucosylation that was mediated by fucosyltransferase 8 in metastatic melanoma [16]; another study determined that fucosyltransferase 8 could facilitate invasion and tumor dissemination, in part due to a reduced cleavage of the cell adhesion molecule L1 [17]; and others identified aberrant glycosylation and related molecules as potential therapeutic targets in cancer treatment, because a disaccharide-based inhibitor of glycosylation could attenuate metastatic melanoma cell dissemination [18].

Since glycosylation may play an important role in the progression and metastasis of melanoma, we hypothesize that genetic variants of glycosylation pathway genes are associated with survival in CM patients. Therefore, we tested this hypothesis by using genotyping data from publicly available melanoma GWAS datasets.

## 2. Results

### 2.1. Patient Features

The discovery dataset included 858 CM patients from The University of Texas MD Anderson Cancer Center (MDACC), and the replication dataset included 409 CM patients from the Nurses’ Health Study (NHS) and Health Professionals Follow-up Study (HPFS); and the baseline characteristics of these patients have been described elsewhere [19,20]. In the MDACC discovery dataset, CM patients were between 17 and 94 years old at diagnosis (52.4 ± 14.4 years old), with a median follow-up time of 81.1 months. There were more males (496, 57.8%) than females (362, 42.2%) and many more diagnosed with stages I/II (709, 82.6%) than with stages III/IV (only one stage IV case) (149, 17.4%). In the NHS/HPFS replication dataset, patients’ age ranged between 34 and 87 years at diagnosis (61.1 ± 10.8 years old), and 66.3% (271) were females. These patients experienced a comparatively longer median follow-up time (179.0 months). The death rates, however, were similar between the MDACC (95/858, 11.1%) and NHS/HPFS (48/409, 11.5%) datasets (Table S1). As none of the principal components were significantly associated with CM survival, no noticeable population stratification in either the MDACC or NHS/HPFS datasets was found; therefore, we did not adjust for these principal components in either the discovery or replication analyses.

### 2.2. Associations between SNPs in the Glycosylation-Related Pathway Genes and CM Disease-Specific Survival (CMSS)

**Figure 1:**
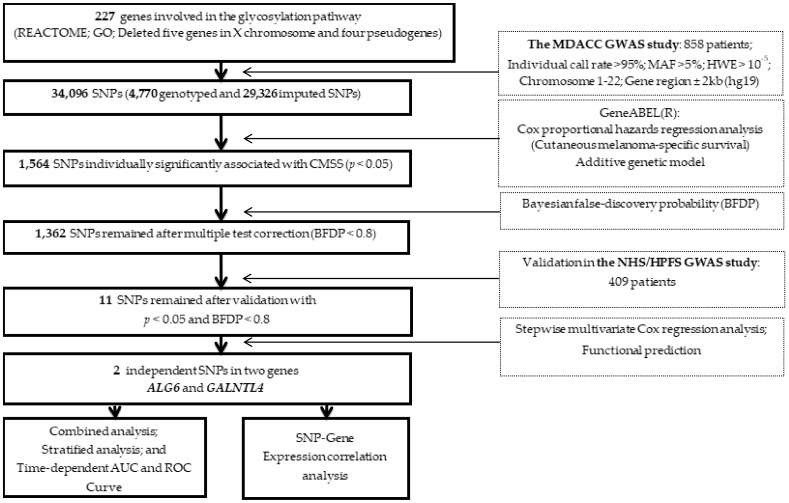
A flow chart of the study design for the selected Single-nucleotide polymorphisms (SNPs) in the glycosylation pathway-related genes.

Figure 1 shows the flowchart of the present study design. We first performed a single-locus analysis for associations of 4,770 genotyped and 29,326 imputed SNPs in the 227 glycosylation pathway genes with CMSS. We found that 1,564 SNPs were associated with CMSS (*p* < 0.05) in an additive genetic model. After multiple test correction by Bayesian false discovery probability (BFDP) < 0.8, 1362 SNPs remained noteworthy; after subsequent replication in the NHS/HPFS dataset, 11 SNPs in five genes remained significantly associated with CMSS. These 11 newly identified SNPs remained statistically significant in the meta-analysis of the two datasets without obvious heterogeneity (Table 1).

### 2.3. Independent SNPs to be Associated with CMSS

**Figure 2:**
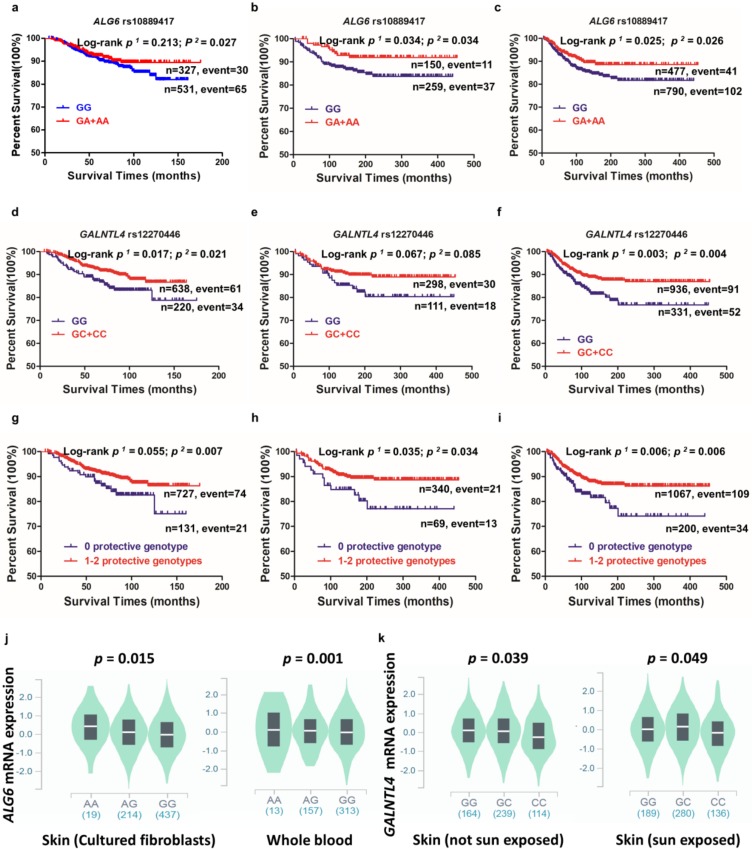
Association of two independent SNPs in glycosylation-related pathway genes with CMSS and their genotypes. Assuming the dominant model was used in the (**a**) MDACC, (**b**) NHS/HPFS, and (**c**) MDACC and NHS/HPFS combined dataset, the Kaplan–Meier survival curve of CMSS with *ALG6* rs10889417 stratification. Assuming the dominant model was used in (**d**) MDACC, (**e**) NHS/HPFS, and (**f**) the MDACC and NHS/HPFS combined dataset, the Kaplan–Meier survival curve of CMSS with *GALNTL4* rs12270446 stratification. The combined risk genotypes on CMSS (Kaplan–Meier survival curves): the dichotomized 0 NPG (Number of Protective Genotype) group and 1-2 NPG group in (**g**) MDACC, (**h**) NHS/HPFS, and (**i**) the MDACC and NHS/HPFS combined dataset. (**j**) The correlation between *ALG6* rs10889417 genotypes with its mRNA expression levels in both cultured skin fibroblasts and whole blood cells from the GTEx (Genotype-Tissue Expression) database. (**k**) The correlations of *GALNTL4* rs12270446 genotypes with its mRNA expression levels in skin tissues from the GTEx database.

We included the 11 significant SNPs, together with other covariates, in a stepwise multivariable Cox regression analysis for the MDACC dataset. We found that five SNPs (rs10889417, rs672748, rs13297246, rs12270446 and rs7287710) in five genes (*ALG6*; *GALNT10* polypeptide N-acetylgalactosaminyltransferase 10; *B4GALT1* beta-1,4-galactosyltransferase 1; *GALNTL4*; and *LARGE* LARGE xylosyl- and glucuronyltransferase 1) remained significantly associated with CMSS (*p* < 0.05). Then, we expanded the model by further including 40 previously reported significant survival-associated SNPs from the MDACC GWAS dataset; two of the newly identified SNPs (*ALG6* rs10889417 and *GALNTL4* rs12270446) remained independent and significantly associated with CMSS (Table 2). Specifically, we observed a significant protective effect of the *ALG6* rs10889417 A allele (*P*_trend_ = 0.023) and the *GALNTL4* rs12270446 C allele (*P*_trend_ = 0.015) on CMSS. These effects were also replicated in the NHS/HPFS dataset (*P*_trend_ = 0.032 and 0.020, respectively) and in the combined MDACC and NHS/HPFS datasets (*P*_trend_ = 0.024 and 0.001, respectively) (Table 3). Kaplan–Meier survival curves were plotted to visually show the associations between CMSS and the genotypes of *ALG6* rs10889417 and *GALNTL4* rs12270446, respectively (Figure 2 a–f). The results of all the selected SNPs are also summarized in a Manhattan plot (Figure S1). Figure S2 provides the quantile–quantile plot of all the SNPs we used. The regional association plot for each of the two independent and statistically significant SNPs is shown in Figure S3. By using the versatile gene-based association study (VEGAS) method, we performed the gene-based test and identified 7 of 221 genes as having an empirical *p* value <0.05. Although no significant gene-based test statistic was found for *ALG6* and *GALNTL4*, the top SNPs in both of these two genes had a significant *p* value <0.05 (Table S2).

### 2.4. Survival of CM Patients with Combined Protective Genotypes

To substantiate the associations between the genotypes of the two independent SNPs and CMSS, we combined the protective genotypes of *ALG6* rs10889417 GA+AA and *GALNTL4* rs12270446 GC+CC into one variable. Patients were divided into three groups according to the number of protective genotypes (NPGs), and the trend test for a dose–response effect of NPG was statistically significant. Specifically, after adjustment for covariates, wherever appropriate, the effect on CMSS was statistical associated with an increased NPG in the MDACC dataset (*P*_trend_ = 0.001), the NHS/HPFS dataset (*P*_trend_ = 0.005), and the MDACC and NHS/HPFS combined dataset (*P*_trend_ = 0.0003) (Table 3).

Patients were also dichotomized into 0 and 1-2 NPG groups. As illustrated in Table 3, we found that the 1-2 NPG group had a greater protective effect on CM death in the MDACC dataset than the 0 NPG group. Conversely, patients who did not have these protective genotypes had a much worse survival in the MDACC dataset (hazards ratio (HR)_adj_ = 1.98; 95% confidence interval (CI) = 1.21–3.26, *p* = 0.007), the NHS/HPFS dataset (HR_adj_ = 1.99; 95% CI = 1.05–3.77, *p* = 0.034), and the combined datasets (HR_adj_ = 1.72; 95% CI = 1.17–2.52, *p* = 0.006). We also used Kaplan–Meier survival curves to visually show the associations between the NPG and CMSS (Figure 2g–i).

### 2.5. Stratified Analysis for Combined Protective Genotypes’ Effect on CMSS

Next, the stratified analysis was performed to test whether the combined effect of the protective genotypes on CMSS was modified by clinicopathological covariables, including age, sex, Breslow thickness, tumor stage, mitotic rate, and ulceration in the MDACC dataset, but only age and sex in the NHS/HPFS dataset. Compared with those with 0 NPG, patients with 1-2 NPG had a significantly better survival, particularly evident in the subgroups aged >60 years, female, regional/distant metastasis, Breslow thickness >1 mm, no ulceration, and mitotic rate >1 in the MDACC dataset and the subgroup aged >60 years in the NHS/HPFS dataset. However, there were no significant interactions between these strata (*p* > 0.05 for all strata, Table S3).

### 2.6. Receiver Operating Characteristic (ROC) Curve and Internal Replication

To further evaluate predictive effects of the two independent SNPs, the time-dependent area was generated using the area under receiver curve (AUC) function of the ROC curve for CM patients in the MDACC and NHS/HPFS datasets in the presence of other covariables. In the MDACC dataset, the predictive performance of 5-year CMSS ROC was modified by the risk genotypes that were added to the model, with the AUC increasing from 65.88% to 72.01% with other covariables (age and sex) as classifiers; however, the change was not statistically significant (*p* = 0.821) (Figure S4b). In the NHS/HPFS dataset, the predictive performance of 5-year CMSS ROC was modified by protective genotypes added to the model, with the AUC increasing from 54.05% to 67.26%, with other covariates (age and sex) as classifiers, and the change was statistically significant (*p* = 0.031) (Figure S4c,d).

### 2.7. In Silico Functional Validation

With the online tools for predicting putative functions of genetic variants, we found that the rs10889417 A allele was significantly associated with increased expression levels of *ALG6* mRNA in cultured skin fibroblasts (*p* = 0.015, Figure 2j) and whole blood cells (*p* = 0.001, Figure 2j). Besides, the *GALNTL4* rs12270446 C allele was correlated with lower mRNA expression levels in normal tissue samples from 517 donors’ suprapubic skin and 605 donors’ sun-exposed lower leg skin from the genotype-tissue expression (GTEx) project (all *p* < 0.05, Figure 2k). Additionally, we also performed expression quantitative trait loci (eQTL) for the correlations between corresponding mRNA expression levels and genotypes of SNPs of the same gene in 373 normal lymphoblastoid cell lines from the 1000 Genomes Project database; however, we did not find any evidence for such a correlation (Figure S5).

The functional prediction was then performed for the two independent SNPs by the online bioinformatics tools SNPinfo [21], RegulomeDB [22], and Haploreg [23] in order to predict their functions, the results of which are summarized in Table S4. Based on experimental data from the ENCODE project available for the 227 glycosylation pathway genes (Table S5), the rs10889417 is located on the Pitx2 motif (Figure S6). By using the PROMO online program, we also found that *ALG6* was a potential target gene of the transcription factor Pitx2 (Table S6). To clarify the effect of gene expression on the survival of cancer patients, we used an online tool based on the TCGA dataset to compare the survival of patients by the cuff-values at the 30th upper percentile and 30th lower percentile of the corresponding gene. We found that the expression levels of *ALG6* seemed not to be substantially associated with melanoma survival (*p* = 0.671) (Figure S7a). On the other hand, the higher expression levels of *GALNTL4* seemed to be associated with a poorer melanoma survival, but the correlation was also not statistically significant (*p* = 0.084) (Figure S7b). However, the higher expression levels of *ALG6* were associated with a better survival in colon adenocarcinoma (*p* = 0.028) and lung squamous cell carcinoma patients (*p* = 0.038), respectively (Figure S7c,e). Meanwhile, the higher expression levels of *GALNTL4* seem to be associated with a poor survival probability in colon adenocarcinoma (*p* = 0.385) and stomach adenocarcinoma patients (*p* = 0.088), respectively; however, the correlation was also not statistically significant (Figure S7d,f).

### 2.8. Mutation Analyses

The mutation status of *ALG6* and *GALNTL4* in CM tissues was assessed using the cBioPortal database for Cancer Genomics. As shown in Figure S8, *ALG6* had a relatively low somatic mutation rate in CM (2.56%, 1/39; 1.28%, 1/78; 0.68, 1/147 and 0.63%, 1/479) in the SKCM UCLA [24], Broad 2014 [25], Yale [26], and TCGA studies [27], respectively. In contrast, *GALNTL4* had a relatively higher somatic mutation rate in CM (3.85%, 1/26; 2.72%, 4/147; 1.65, 2/121, 1.88%, 9/479 and 1.28%, 1/78) in the Broad DFI [28], Yale [26], Broad 2012 [29] TCGA, and Broad 2014 studies [25], respectively. Considering that there are few mutations in these two genes, our results indicated that the functional SNPs in *ALG6* may play relatively important roles in the dysregulated mRNA expression in tumor tissues, and the mutation may also play a role in the functional change and expression of *GALNTL4* in addition to the causal SNPs.

## 3. Discussion

In the present study, we investigated the associations between 34,096 genetic variants of 227 glycosylation pathway genes and CMSS by using a two-phase analysis of genotyping data from two previously published GWAS datasets: A hospital case-control dataset for the discovery and a cohort follow-up dataset for the replication. We found that two SNPs (i.e., *ALG6* rs10889417G>A and *GALNTL4* rs12270446G>C) were independently associated with the survival of CM patients. In subsequent genotype–mRNA expression correlation analysis, we found that the low death-risk-associated rs10889417 A allele was associated with increase in *ALG6* mRNA expression levels in cultured skin fibroblasts and whole blood cells and that the rs12270446 G allele was associated with decrease in *GALNTL4* mRNA expression levels in skin tissues.

Since about half of human proteins are glycoproteins, and glycosylation is one of the most important post-transcriptional modifications, it is undeniable that glycosylation of different proteins plays a key role in multiple cellular activities, including tumor proliferation, invasion, metastasis, tumor-induced immune regulation, and drug resistance [10]. For example, the biological function of integrin is related to the adhesion of the extracellular matrix, and integrin is also found to be one of the abundant proteins in metastatic melanoma cells [30]. One study has identified that integrin-reduced cell adhesion is the result of modification of N-acetylglucosaminyltransferase III, which is involved in the N-glycosylation process [31].

Although the present study suggested that the expression of *ALG6* seemed not to have a significant effect on CM survival, a high *ALG6* expression was associated with a better overall survival (OS) in colon adenocarcinoma and lung squamous cell carcinoma patients. In a previous animal study, however, we noted that knockdown of the mouse *Alg6* gene increased melanoma lung metastasis without affecting primary tumor growth, which may lead to a shorter survival [32]. However, the gene expression profiles in tumor tissues are more likely affected by mutations in the driver genes commonly seen in tumor tissues. Therefore, further epidemiological investigation and tissue-based functional experiments are needed to clarify these discrepancies in the future.

Mutations in *ALG6* may lead to congenital disorders of glycosylation (CDG), called ALG6-CDG, of which missense mutations P. A333V and P. I299Del are the most common mutations [33]. Some patients with CDG have severe immune deficiencies, likely leading to tumorigenesis [34]. For example, in acquired immunodeficiency patients with CM, such as those with human immunodeficiency virus infection, tumors may have explosive progress [35] while in the melanoma immune response, the inflammation and tumor immune response have shown significant abnormalities, leading to the progress of the disease [36,37]. These suggest that a possible effective treatment for CM may be immunotherapy, which has recently become important in improving the prognosis of melanoma patients [38]. Although the *ALG6* gene malfunction may play a role in the poor prognosis of melanoma, we also showed that the mutation rate of *ALG6* in melanoma tissues was less than 2.56%, and thus the overall expression levels of *ALG6* in melanoma tissues are more likely to be affected by SNPs. Further supporting evidence is that *ALG6* rs10889417 is located on the Pitx2 motif, indicating its potential regulatory roles in *ALG6* mRNA expression. Because of the close relationship established between *ALG6* and N-linked glycosylation and melanoma cell activity [39], genetic variation in *ALG6* is likely to play a role in CM progression and prognosis.

No published study has investigated the role of *GALNTL4* in CM tumor progression and survival. However, previous GWAS studies have shown an association between genetic variants of *GALNTL4* and protective effects against differentiated thyroid cancer [40], and *GALNTL4* SNPs are reportedly associated with the cisplatin sensitivity of urothelial cancer [41] and the efficacy of gemcitabine combined with platinum in the chemo treatment of bladder urothelial carcinoma [42]. In the present study, we showed that the rs12270446 G>A located in the intron of *GALNTL4* was associated with CMSS. Given the fact that the mutation rate of *GALNTL4* in melanoma tumors is low, it is likely that genetic variant-associated *GALNTL4* gene expression may be the mechanism underlying the observed association, which deserves further investigation.

The present study is subject to several limitations. First, the two GWAS datasets we employed were both from Caucasian populations, which may not be generalizable to the general population. Second, all the participants in the two GWAS studies may have been treated with distinctive therapies that were not made available for our analysis. However, we believe these therapy regimens, if any, might not have been selective to the genetic variation of the patients. Third, we obtained the glycosylation-related genes from the GSEA/MSigDB website, a major publicly recognized dataset, but we might have missed some other unknown and important genes involved in this metabolic pathway. Fourth, the literature has well documented that the prognosis of stage III/IV melanoma is significantly different. Unfortunately, we had only one case of stage IV CM cases in the MDACC dataset. This made it impossible for us to perform stratified analysis by stages III and IV separately. Lastly, we were not able to investigate the biological mechanisms to understand how *ALG6* rs10889417 G>A and *GALNTL4* rs12270446 G>C influence CMSS, which should be further investigated in the future.

## 4. Materials and Methods

### 4.1. Study Populations

The discovery analysis used genotyping data from the MDACC melanoma GWAS study, and the replication analysis used genotyping data of the GWAS dataset from NHS/HPFS. In the MDACC study, all patients from a hospital-based case-control CM study were recruited among non-Hispanic white patients. In the NHS/HPFS study, patients were those who developed CM during the course of follow-up. Detailed descriptions of the subject selection and data collection for these two GWASs have been published elsewhere [19,20]. According to the protocol approved by the Institutional Review Boards of the MDACC, Brigham and Women’s Hospital, and Harvard T.H. Chan School of Public Health, all subjects provided a written informed consent and the registration form for participation as required.

### 4.2. Gene Selection and SNP Genotyping

We selected 227 glycosylation pathway genes located on the autosomes by inquiring the Molecular Signatures Database of the GSEA website [43,44] (Table S5). Genomic DNA was extracted from whole blood cells in the MDACC GWAS dataset and used for genotyping by the Illumina HumanOmni-Quad_v1_0_B array. The National Center for Biotechnology Information Database of Genotypes and Phenotypes (dbGaP Study Accession: phs000187.v1.p1) provided the genotyping data. Based on the 1000 Genomes Project, phase I v2 CEU (March 2010 release), utilizing the MACH software, we performed the genome-wide imputation. We included both typed (having a genotyping success rate of 95% with a Hardy–Weinberg equilibrium *p* value of 10^-5^) and imputed (*r*^2^ > 0.8) common SNPs (a minor allele frequency of 0.05) within ±2 kilobase flanking regions of these glycosylation pathway genes. In the NHS/HPFS GWAS dataset, whole blood DNA samples were used for genotyping with the HumanHap610 array, Affymetrix 6.0 array, and Illumina HumanHap550 array, and imputation was based on the haplotype information and genotyped SNPs from phase II HapMap CEU data (March 2012 release), applying the program (MACH March 2012 release) using a similar quality control to that for the MDACC GWAS dataset. For each glycosylation-related SNP, quantile–quantile and Manhattan plots were generated to summarize the genome-wide meta-analysis.

### 4.3. Statistical Methods

In the MDACC discovery analysis using a multivariable Cox proportional hazards regression model, we first assessed in a single-locus analysis the associations between selected SNPs in 227 glycosylation pathway genes and CMSS by calculating HR and its 95% CI using R software (GenABEL package) [45]. After being adjusted for other covariaales, including age, sex, Breslow thickness, ulceration, tumor stage, and mitotic rate, the multivariable analysis was performed. In the NHS/HPFS replication analysis, however, the only covariaales available for adjustments were age and sex. Survival time was defined as the time between the dates of diagnosis of CM to the date of death. Patients known to be alive were censored at the time of the last contact.

Since most SNPs were estimated with a high level of linkage disequilibrium (LD), we utilized BFDP with a threshold of r^2^ =0.8 for LD in multiple test corrections, as recommended [40], rather than the false discovery rate. We also used a prior probability of 0.1 to detect an HR of 2.0, which was related to the variant genotype or minor allele of SNP (*p* < 0.05). Next, in performing stepwise multivariable Cox regression analysis with those validated SNPs for selecting independent tagging SNPs, we used the MDACC dataset that had more detailed covariate information. We then conducted a meta-analysis to combine the estimates from the MDACC dataset with those in the NHS/HPFS dataset using PLINK 1.90 with the Cochran’s Q statistics and *I*^2^. A fixed-effects model was used, because no significant heterogeneity was found between the two datasets (Q test *p* > 0.1 and *I*^2^ < 25.0%). We also performed gene-based tests by using the VEGAS approach that was integrated in the VEGAS2 program [46,47]. In brief, for a given gene with n low LD SNPs, the correlation *p* value was first transformed into a Chi-squared statistic with one degree of freedom. Then, the gene-based test statistics were calculated by adding all Chi-squared statistics within the gene. A large number of simulations were carried out with multivariable normal distribution, and the proportion of simulated test statistics based on the empirical *p*-value of gene is more than that of observed test statistics based on the gene. Kaplan–Meier estimation of the survival functions and log-rank test was carried out, and the comprehensive effect of the visual protective genotype was taken as a genetic score for predicting CMSS.

ROC curves were further constructed to illustrate the ability of the time-dependent AUC to predict CMSS. The ROC plots were generated by using the R packages “survival” and “timeROC” [48]. For the stratified analyses by subgroup, we calculated the inter-study heterogeneity and assessed the interaction between strata. For the significant and independent SNPs identified from the multivariable analysis, functional prediction was performed by using bioinformatics online tools: RegulomeDB [22,49], SNPinfo [21,50], and HaploReg [23,51]. The transcription factors in the promoter regions were predicted with PROMO online tools [52].

By using R (version 3.5.0) software for linear regression analysis, the eQTL were further analyzed to evaluate the correlation between SNPs and the mRNA expression levels of the genes. The mRNA expression data of these genes were obtained from 373 lymphoblastic cell lines of European descent included in the 1000 genome project [43] and GTEx project version 8 [44]. Next, we evaluated the associations of mRNA expression with the OS of additional cancers through Kaplan–Meier analysis in colon adenocarcinoma, lung squamous cell carcinoma, and stomach adenocarcinoma patients [53]. Unless otherwise specified, all statistical analyses were carried out using SAS software (version 9.4; SAS Institute, Cary, NC).

## 5. Conclusions

Two independent SNPs (i.e., *ALG6* rs10889417G>A and *GALNTL4* rs12270446G>C) were found to be significantly associated with CMSS in the MDACC discovery and NHS/HPFS replication datasets. The combined analysis showed that these two SNPs were significantly associated with survival, and patients’ better prognosis may be achieved through more protective genotypes influencing gene expression. Our findings provide some new insights for further functional studies to identify potential molecular mechanisms underlying the observed CM survival.

## Figures and Tables

**Table 1:** Meta-analysis of 11 validated SNPs in the glycosylation pathway genes using two independently published melanoma genome-wide association study (GWAS) datasets.

SNP	Allele ^1^	Gene	Discovery-MDACC (n = 858)				Validation-NHS/HPFS (n = 409)				Combined-Meta-Analysis (n = 1267)			
			EAF	HR (95% CI)	*p* ^2^	BFDP	EAF	HR (95% CI)	*p* ^3^	BFDP	*P*_het_	I2	HR (95% CI)	*p* ^4^
rs78409522 ^$^	C > T	*B4GALT1*	0.08	2.12 (1.32–3.42)	0.002	0.389	0.06	2.34 (1.27–4.29)	0.006	0.616	0.802	0	2.20 (1.51–3.20)	3.59 × 10^−5^
rs16918998 ^#^	T > C	*B4GALT1*	0.08	2.12 (1.32–3.42)	0.002	0.389	0.06	2.34 (1.27–4.29)	0.006	0.616	0.802	0	2.20 (1.51–3.20)	3.59 × 10^−5^
rs13297246 ^$^	G > A	*B4GALT1*	0.16	1.83 (1.32–2.52)	2.46 × 10^−4^	0.07	0.16	1.85 (1.15–3.00)	0.012	0.555	0.971	0	1.84 (1.05–2.40)	8.43 × 10^−6^
rs2183124 ^$^	G > A	*B4GALT1*	0.08	2.08 (1.34–3.24)	0.001	0.288	0.06	2.11 (1.17–3.79)	0.013	0.693	0.97	0	2.09 (1.47–2.98)	4.23 × 10^−5^
rs10971414 ^$^	C > T	*B4GALT1*	0.08	2.08 (1.34–3.24)	0.001	0.288	0.06	2.07 (1.15–3.75)	0.016	0.725	0.99	0	2.08 (1.46–2.96)	5.14 × 10^−5^
rs12270446 ^$^	G > C	*GALNTL4*	0.5	0.69 (0.52–0.93)	0.015	0.65	0.48	0.61 (0.40–0.93)	0.02	0.716	0.637	0	0.66 (0.52–0.84)	0.004
rs7128890 ^#^	A > G	*GALNTL4*	0.39	1.39 (1.03–1.87)	0.033	0.756	0.41	1.54 (1.04–2.28)	0.031	0.758	0.684	0	1.44 (1.14–1.83)	0.003
rs10889417 ^#^	G > A	*ALG6*	0.21	0.64 (0.43–0.94)	0.023	0.719	0.21	0.52 (0.29–0.95)	0.032	0.791	0.566	0	0.60 (0.44–0.83)	0.002
rs672748 ^$^	A > G	*GALNT10*	0.19	1.45 (1.04–2.03)	0.03	0.756	0.19	1.82 (1.17–2.85)	0.008	0.597	0.604	0	1.71 (1.17–2.51)	0.006
rs12628567 ^$^	C > T	*LARGE*	0.12	1.50 (1.02–2.22)	0.042	0.794	0.12	1.83 (1.07–3.12)	0.026	0.758	0.556	0	1.61 (1.17–2.20)	0.003
rs7287710 ^#^	T > C	*LARGE*	0.12	1.50 (1.02–2.22)	0.042	0.794	0.13	1.80 (1.05–3.07)	0.031	0.774	0.59	0	1.60 (1.17–2.19)	0.001

^1^ Reference allele/effect allele; ^2^ Adjusted for age, sex, Breslow thickness, distant/regional metastasis, ulceration, and mitotic rate in the additive model; ^3^ Adjusted for age and sex in the additive model; ^4^ Meta-analysis in the fixed-effect model; ^$^ Imputed SNP; ^#^ Genotyped SNP.

**Table 2:** Two independent SNPs in a stepwise multivariable Cox regression analysis with adjustment for other covariates and previous published SNPs in The University of Texas MD Anderson Cancer Center (MDACC) dataset.

Parameter	Category ^1^	Frequency	HR (95% CI)^2^	*p* ^2^	HR (95% CI) ^3^	*p* ^3^
Age	≤50/>50	371/487	1.02 (1.01–1.04)	0.011	1.05 (1.02–1.07)	<0.0001
Sex	Female/Male	362/496	1.30 (0.81–2.10)	0.275	1.24 (0.74–2.09)	0.415
Regional/distant metastasis	No/Yes	709/149	3.75 (2.43–5.77)	<0.0001	12.22 (6.70–22.29)	<0.0001
Breslow thickness (mm)	≤1/>1	347/511	1.16 (1.10–1.22)	<0.0001	1.26 (1.17–1.36)	<0.0001
Ulceration	No/Yes	681/155	3.12 (2.00–4.88)	<0.0001	4.93 (2.84–8.54)	<0.0001
Mitotic rate (mm^2^)	≤1/>1	275/583	2.83 (1.34–5.96)	0.006	2.18 (0.94–5.08)	0.07
*ALG6* rs10889417 G>A	GG/GA/AA	531/293/34	0.62 (0.42–0.92)	0.016	0.48 (0.29–0.78)	0.003
*GALNTL4* rs12270446 G>C	GG/GC/CC	220/418/220	0.61 (0.45–0.82)	0.001	0.61 (0.43–0.88)	0.007

^1^ The “category/” was used as the reference. ^2^ Stepwise multivariable Cox analysis included age, sex, regional/distant metastasis, Breslow thickness, ulceration, mitotic rate and SNPs; ^3^ The 40 published SNPs were adjusted for post-stepwise analysis. The 40 SNPs were reported in previous publications (PMID: 25953768, 25628125, 25243787, 26575331, 30734280, 30596980, 29313974, 29088810, 28796414, 28542949, 28499756, 27914105 and 27578485).

**Table 3:** Associations between two independent SNPs in the glycosylation-related genes and CMSS of patients in the MDACC dataset, the NHS/HPFS dataset, and the MDACC and NHS/HPFS combined dataset.

	MDACC (n = 858)				NHS/HPFS (n = 409)				MDACC + NHS/HPFS (n = 1,267)			
Genotype	Frequency		Multivariable Analysis ^1^		Frequency		Multivariable Analysis ^2^		Frequency		Multivariable Analysis ^3^	
	All	Death (%)	HR (95%CI)	*p*	All	Death (%)	HR (95%CI)	*p*	All	Death (%)	HR (95%CI)	*p*
***ALG6* rs10889417 G > A**												
GG	531	65 (12.2)	1.00		259	37 (14.3)	1.00		790	102 (12.9)	1.00	
GA	293	27 (9.2)	0.64 (0.40–1.02)	0.059	128	10 (7.8)	0.52 (0.26–1.05)	0.068	421	37 (8.8)	0.68 (0.47–1.00)	0.047
AA	34	3 (8.8)	0.42 (0.13–1.34)	0.142	22	1 (4.6)	0.28 (0.04–2.02)	0.206	56	4 (7.1)	0.51 (0.19–1.40)	0.192
Trend test				0.023				0.032				0.024
GA+AA	327	30 (9.2)	0.60 (0.38–0.94)	0.027	150	11 (7.3)	0.48 (0.25–0.95)	0.034	477	41 (8.6)	0.66 (0.46–0.95)	0.026
***GALNTL4* rs12270446 G > C**												
GG	220	34 (15.5)	1.00		111	18 (16.2)	1.00		331	52 (15.7)	1.00	
GC	418	43 (10.3)	0.66 (0.42–1.05)	0.079	207	25 (12.1)	0.73 (0.40–1.34)	0.304	625	68 (10.9)	0.68 (0.48–0.98)	0.038
CC	220	18 (8.2)	0.49 (0.27–0.89)	0.02	91	5 (5.5)	0.32 (0.12–0.86)	0.023	311	23 (7.4)	0.46 (0.28–0.75)	0.002
Trend test				0.015				0.02				0.001
GC+CC	638	61 (9.6)	0/60 (0.39–0.93)	0.021	298	30 (10.1)	0.60 (0.33–1.07)	0.085	936	91 (9.7)	0.61 (0.43–0.86)	0.004
**Number of protective genotypes ^4^**												
0	131	21 (16.0)	1.00		69	13 (18.8)	1.00		200	34 (17.0)	1.00	
1	489	57 (11.7)	0.59 (0.36–0.99)	0.046	232	29 (12.5)	0.62 (0.32–1.19)	0.15	721	86 (11.9)	0.68 (0.46–1.01)	0.058
2	238	17 (7.14)	0.32 (0.16–0.63)	0.001	108	6 (5.6)	0.26 (0.10–0.70)	0.007	346	23 (6.65)	0.38 (0.22–0.65)	0.0003
Trend test				0.001				0.005				0.0003
1-2	727	74 (10.2)	1.00		340	35 (10.3)	1.00		1067	109 (10.22)	1.00	
0	131	21 (16.0)	1.98 (1.21–3.26)	0.007	69	13 (18.8)	1.99 (1.05–3.77)	0.034	200	34 (17.0)	1.72 (1.17–2.52)	0.006

^1^ Age, sex, Breslow thickness, distant/regional metastasis, ulceration, and mitotic rate were adjusted in the MDACC dataset; ^2^ Age and sex were adjusted in the NHS/HPFS dataset; ^3^ Age and sex were adjusted in the combined MDACC and NHS/HPFS dataset; ^4^ Protective genotypes include *ALG6* rs10889417 GA+AA and *GALNTL4* rs12270446 GC+CC.

## Supplementary Materials

The following materials are available online at https://www.mdpi.com/2072-6694/12/2/288/s1, Table S1: Distributions of characteristics of CM patients in MDACC and NHS/HPFS; Table S2: Significant results of glycosylation related genes in the gene-based test with the VEGAS method; Table S3: Stratified analysis of protective genotypes of the identified SNPs in the MDACC and NHS/HPFS datasets; Table S4: Function prediction of two independent SNPs in *GALNTL4* and *ALG6;* Table S5: List of 227 selected glycosylation pathway genes. Figure S1: Manhattan plots of associations between SNPs and CMSS in the MDACC dataset (a) and the NHS/HPFS dataset (b). Figure S2: Quantile–quantile plot of all SNPs in the MDACC GWAS dataset. Figure S3: Regional association plots contained 100-kb up and downstream of the gene regions in (a) *ALG6* and (b) *GALNTL4*. Figure S4: Time-dependent AUC and ROC curves for five-year CMSS prediction. Figure S5: Associations between genotypes and their corresponding mRNA expression levels. Figure S6: Functional prediction of SNPs in the ENCODE project. Figure S7: Kaplan-Meier analysis for cancer patients by expression levels of *ALG6* and *GALNTL4*; and Figure S8: Mutation analysis of *ALG6* and *GALNTL4* gene in cutaneous melanoma tumor tissues by using public available data in the database of the cBioportal for Cancer Genomics (http://www.cbioportal.org).
