# Supplementary material for: Novel Genetic Variants of ALG6 and GALNTL4 of the Glycosylation Pathway Predict Cutaneous Melanoma-Specific Survival
Source: Cancers (Basel). 2020 Jan 24;12(2):288. doi: 10.3390/cancers12020288 (PMC7072252; doi:10.3390/cancers12020288)
Supplement: Supplementary file 1 [file cancers-12-00288-s001.pdf]

Supplementary Table S1. Distributions of characteristics of CM patients in MDACC and NHS/HPFS datasets

Parameter		Frequency		MFT	HR (95% CI)[1]	*P*[1]
		Patient	Death (%)			
MDACC		858	95 (11.1)	81.1		
Age (years)	≤50	371	31 (8.4)	85.8	1.00	
	>50	487	64 (13.1)	78.1	1.69 (1.10-2.59)	0.017
Sex	Female	362	26 (7.2)	85.9	1.00	
	Male	496	69 (13.9)	77.8	2.07 (1.32-3.25)	0.002
Stage	Stage I/II	709	51 (7.2)	82.7	1.00	
	Stage III/IV	149	44 (29.5)	69.4	4.78 (3.19-7.15)	<0.001
Breslow thickness (mm)	≤1	347	7 (2.0)	85.0	1.00	
	>1	511	88 (17.2)	78.1	9.17 (4.25-19.80)	<0.001
Ulceration	No	681	48 (7.1)	84.0	1.00	
	Yes	155	43 (27.7)	64.3	4.91 (3.29-7.42)	<0.001
	Missing	22				
Mitotic rate (mm^2)	≤1	275	9 (3.3)	82.2	1.00	
	>1	583	86 (14.8)	80.1	4.67 (2.35-9.29)	<0.001
NHS/HPFS		409	48 (11.5)	179.0		
Age (years)	≤50	72	3 (4.2)	352.5	1.00	
	>50	337	45 (13.4)	167.0	4.04 (1.25-13.06)	0.020
Sex	Female	271	31 (11.4)	198.0	1.00	
	Male	138	17 (12.3)	155.5	1.16 (0.64-2.10)	0.622

Abbreviations: CM, cutaneous melanoma; MDACC, The University of Texas MD Anderson Cancer Center; NHS/HPFS, the Nurses' Health Study/Health Professionals Follow-up Study; MFT, median follow-up time (months); HR, hazards ratio; CI, confidence interval;

[1]Univariable Cox proportional hazards regression analysis

Supplementary Table S2. Significant results of glycosylation related genes in the gene-based test with the VEGAS method

Chr	Gene	nSNPs	nSims	Start position	Stop position	Test[1]	Gene *P*-value	Best-SNP	Best-SNP *P*-value
15	*ADAMTS7*	47	1.00E+05	79049544	79105773	239.567	0.006	rs12903203	0.002
3	*GMPPB*	6	1.00E+05	49756908	49763407	40.638	0.007	rs1049256	0.007
9	*B4GALT1*	156	1.00E+05	33108638	33169356	592.399	0.01	rs13297246	0.0002
14	*POMT2*	107	1.00E+05	77739298	77789225	316.825	0.013	rs12431465	0.005
21	*ADAMTS5*	120	1.00E+05	28288230	28341439	382.795	0.019	rs162506	0.003
9	*POMT1*	31	1.00E+05	134376288	134401193	108.581	0.024	rs117287406	0.007
20	*SLC35C2*	16	1.00E+05	44976166	44995097	48.589	0.037	rs12480667	1.59E-05
11	*GALNTL4*	1266	1000	11290420	11645561	1368.899	0.311	rs10741557	0.001
1	*ALG6*	132	1000	63831260	63906233	49.564	0.827	rs10889417	0.023

[1] Gene-based test statistic

Abbreviations: VEGAS, Versatile Gene-based Association Study; Chr, Chromosome; SNP, single-nucleotide polymorphism; nSNPs, number of SNPs; nSims, number of simulations.

Supplementary Table S3. Stratified analysis of protective genotypes of the identified SNPs in the MDACC and NHS/HPFS datasets

Characteristics	0 NPG[1]		1-2 NPG[1]		Univariate analysis		Multivariate analysis[2]		Interaction[3]
	All	Death (%)	All	Death (%)	HR (95% CI)	*P*	HR (95% CI)	*P*	
**MDACC dataset**									
**Age (years)**									
≤ 60	59	6 (10.2)	312	25 (8.0)	0.75 (0.31-1.82)	0.517	0.90 (0.35-2.31)	0.819	
> 60	72	15 (20.8)	415	49 (11.8)	0.56 (0.31-1.00)	0.05	0.43 (0.23-0.78)	0.005	0.212
**Sex**									
Male	80	13 (16.3)	416	56 (13.5)	0.83 (0.45-1.51)	0.533	0.61 (0.33-1.13)	0.116	
Female	51	8 (15.7)	311	18 (5.8)	0.35 (0.15-0.81)	0.014	0.31 (0.13-0.76)	0.011	0.249
**Regional/distant metastasis**									
No	106	10 (9.4)	603	41 (6.8)	0.73 (0.37-1.46)	0.371	0.61 (0.30-1.24)	0.171	
Yes	25	11 (44.0)	124	33 (26.61)	0.53 (0.27-1.05)	0.07	0.39 (0.19-0.82)	0.012	0.399
**Breslow thickness (mm)**									
≤1	52	1 (1.9)	295	6 (2.0)	1.13 (0.14-9.40)	0.91	1.95 (0.17-22.66)	0.595	
>1	79	20 (25.32)	432	68 (15.7)	0.59 (0.36-0.97)	0.037	0.50 (0.30-0.83)	0.007	0.469
**Ulceration**									
No	100	13 (13.0)	581	35 (6.02)	0.46 (0.24-0.86)	0.016	0.44 (0.23-0.84)	0.013	
Yes	28	8 (28.6)	127	35 (27.56)	0.99 (0.46-2.13)	0.972	0.64 (0.28-1.44)	0.278	0.517
Missing	22								
**Mitotic rate (mm²)**									
≤1	38	1 (2.6)	237	8 (3.4)	1.35 (0.17-10.83)	0.775	1.02 (0.12-8.93)	0.99	
>1	93	20 (21.5)	490	66 (13.5)	0.60 (0.36-0.99)	0.045	0.47 (0.28-0.79)	0.004	0.438
**NHS/HPFS dataset**									
**Age (years)**									
≤ 60	36	5 (13.9)	179	16 (8.94)	0.64 (0.24-1.76)	0.389	0.66 (0.24-1.82)	0.426	
> 60	33	8 (24.2)	161	19 (11.8)	0.42 (0.18-0.96)	0.04	0.42 (0.19-0.97)	0.042	0.164
**Sex**									
Male	23	4 (17.4)	115	13 (11.3)	0.64 (0.21-1.97)	0.438	0.60 (0.19-1.85)	0.37	
Female	46	9 (19.6)	225	22 (9.8)	0.46 (0.21-1.00)	0.051	0.47 (0.22-1.03)	0.058	0.096

[1] NPG = Number of protective genotypes: *ALG6* rs10889417 GA+AA and *GALNTL4* rs12270446 GC+CC;

[2] Adjusted for age, sex, Breslow thickness, distant/regional metastasis, ulceration and mitotic rate in Cox models of SNPs and CMSS in the MDACC dataset and adjusted for age and sex only in the NHS/HPFS dataset;

[3] Interaction: the interaction between the protective genotypes and each clinical variable.

Abbreviations: SNP, single-nucleotide polymorphism; MDACC, The University of Texas MD Anderson Cancer Center; NHS/HPFS, the Nurses' Health Study/Health Professionals Follow-up Study; HR, hazards ratio; CI, confidence interval; *GALNTL4*, polypeptide N-acetylgalactosaminyltransferase L4 ; *ALG6*, ALG6 alpha-1,3-glucosyltransferase.

Supplementary Table S4. Function prediction of two independent SNPs in *GALNTL4* and *ALG6*.

**SNP**	**Gene**	**Chr**	**Position (hg 38)**	**RegDB**[1]	**Haploreg v4.1**[2]					
					**Enhancer histone marks**	**DNAse**	**Motifs changed**	**GRASP QTL hits**	**Selected eQTL hits**	**dbSNP functional annotation**
rs12270446	*GALNTL4*	11	11429816	4	MUS					intronic
rs10889417	*ALG6*	1	63370230	6			6 altered motifs	8 hits	49 hits	intronic

[1] RegulomeDB (http://www.regulomedb.org)

[2] HaploReg v4.1 (http://archive.broadinstitute.org/mammals/haploreg/haploreg.php)

Abbreviations: SNP, single-nucleotide polymorphism; Chr, chromosome; dbSNPfuncannot, dbSNP function annotation; *GALNTL4*, polypeptide N-acetylgalactosaminyltransferase L4 ; *ALG6*, ALG6 alpha-1,3-glucosyltransferase.

Supplementary Table S5. List of 227 genes in the glycosylation pathway

Dataset	Name of pathway [1]	Selected genes	Number of genes
REACTOME	REACTOME_ADVANCED_GLYCOSYLATION_ENDPRODUCT_RECEPTOR_SIGNALING	*AGER, APP, CAPZA1, CAPZA2, DDOST, HMGB1, LGALS3, MAPK1, MAPK3, PRKCSH, S100A12, S100B, SAA1*	13
REACTOME	REACTOME_ASPARAGINE_N_LINKED_GLYCOSYLATION	*ALG1, ALG10, ALG10B, ALG11, ALG12, ALG13, ALG14, ALG2, ALG3, ALG5, ALG6, ALG8, ALG9, B4GALT1, B4GALT2, B4GALT3, B4GALT4, B4GALT5, B4GALT6, CALR, CANX, DAD1, DDOST, DOLK, DOLPP1, DPAGT1, DPM1, DPM2, DPM3, EDEM1, EDEM2, EDEM3, FUT8, GANAB, GFPT2, GMPPA, GMPPB, GNPNAT1, LMAN1, LOC285407, MAN1A1, MAN1A2, MAN1B1, MAN1C1, MAN2A1, MANEA, MCFD2, MGAT1, MGAT2, MGAT3, MGAT4A, MGAT4B, MGAT4C, MGAT5, MLEC, MOGS, MPI, PDIA3, PGM3, PMM1, PMM2, PREB, PRKCSH, RFT1, RPN1, RPN2, SAR1B, SEC13, SEC23A, SEC24B, SEC24C, SEC24D, SEC31A, ST6GAL1, ST8SIA2, ST8SIA3, ST8SIA6, STT3A, TUSC3, UGGT1, UGGT2*	81
REACTOME	REACTOME_O_LINKED_GLYCOSYLATION_OF_MUCINS	*B3GNT2, B3GNT3, B3GNT4, B3GNT5, B3GNT6, B3GNT7, B3GNT8, B3GNT9, B3GNTL1, B4GALT5, C1GALT1, C1GALT1C1, GALNT1, GALNT10, GALNT11, GALNT12, GALNT13, GALNT14, GALNT2, GALNT3, GALNT5, GALNT6, GALNT7, GALNT8, GALNT9, GALNTL1, GALNTL2, GALNTL4, GALNTL5, GALNTL6, GCNT1, GCNT3, GCNT4, GCNT6, LOC652741, LOC732162, MUC12, MUC13, MUC15, MUC16, MUC17, MUC19, MUC2, MUC21, MUC3B, MUC4, MUC5AC, MUC5B, MUC6, MUC7, MUCL1, ST3GAL1, ST3GAL2, ST3GAL3, ST6GAL1, ST6GALNAC2, ST6GALNAC3, ST6GALNAC4, WBSCR17*	59
GO	GO_PROTEIN_N_LINKED_GLYCOSYLATION	*ALG12, ALG5, ALG6, ALG8, B4GALT1, B4GALT7, C20orf173, CCDC126, DAD1, DDOST, DERL3, DOLPP1, DPAGT1, DPM1, DPM2, DPM3, EDEM1, EDEM2, EDEM3, ENTPD5, FUT8, GAL3ST1, GNPTAB, GNPTG, GORASP1, KCNE1, KIAA2018, LMAN1, MAGT1, MAN1A1, MAN1A2, MAN1B1, MAN1C1, MAN2A1, MAN2A2, MCFD2, MGAT2, MGAT3, MGAT4A, MGAT4B, MGAT4C, MGAT5, MGAT5B, MOGS, NUDT14, OSTC, PGM3, PRKCSH, RPN1, RPN2, ST3GAL1, ST3GAL2, ST3GAL3, ST3GAL4, ST3GAL5, ST3GAL6, ST6GAL1, ST6GAL2, ST6GALNAC1, ST6GALNAC2, ST6GALNAC3, ST6GALNAC4, ST6GALNAC5, ST6GALNAC6, ST8SIA2, ST8SIA3, ST8SIA4, STT3A, STT3B, SYVN1, TMEM165, TUSC3, UBE2G2, UBE2J1, VCP*	75

GO	GO_REGULATION_OF_PROTEIN_GLYCOSYLATION	*ACER2, ADNP, ARFGEF1, CHP, FKTN, GOLGA2, IL15, KAT2B, KIAA0020, MT3, OSTBETA, RAMP1, TINF2, TMEM59*	14
GO	GO_PROTEIN_O_LINKED_GLYCOSYLATION	*A4GNT, ADAMTS13, ADAMTS5, ADAMTS7, ADAMTSL1, ADAMTSL4, ALG5, B3GALNT2, B3GALTL, B3GNT1, B3GNT2, B3GNT3, B3GNT4, B3GNT5, B3GNT6, B3GNT7, B3GNT8, B4GALT5, C1GALT1, C1GALT1C1, C3orf39, C3orf64, CFP, CHST4, DAG1, DPM1, DPM2, DPM3, FKRP, FKTN, GALNT1, GALNT10, GALNT11, GALNT12, GALNT13, GALNT14, GALNT2, GALNT3, GALNT4, GALNT5, GALNT6, GALNT7, GALNT8, GALNT9, GALNTL2, GCNT1, GCNT3, GCNT4, GXYLT1, GXYLT2, GYLTL1B, ISPD, KCNE1, LARGE, MUC1, MUC12, MUC13, MUC15, MUC16, MUC17, MUC19, MUC2, MUC20, MUC21, MUC3A, MUC3B, MUC4, MUC5AC, MUC5B, MUC6, MUC7, MUCL1, OGT, PGM3, POFUT1, POFUT2, POGLUT1, POMGNT1, POMT1, POMT2, SDF2, SDF2L1, SGK196, SLC35C2, SPON1, ST3GAL1, ST3GAL2, ST3GAL3, ST3GAL4, ST6GAL1, ST6GALNAC2, ST6GALNAC4, ST8SIA6, TET1, TET2, TET3, THBS1, TMEM5, TRAK1, TRAK2, VEGFB*	101
KEGG	—	—	0
PID	—	—	0
Genes removed		Deleting 109 duplicated genes, 5 genes in X chromosome and 5 pseudogenes	119
Total genes			227

[1] Genes were selected based on online datasets and literatures;

Keyword for MSigDB: Glycosylation

Organism: Homo sapiens

MSigDB = Molecular signatures database (http://software.broadinstitute.org/gsea/index.jsp)

Supplementary Table S6. Function prediction of the validated SNPs in *ALG6 and GALNTL4*

SNP	Gene	Chr	Position (hg 38)	RegDB1	Haploreg v4.12					
					Enhancer histone marks	DNAse	Motifs changed	GRASP QTL hits	Selected eQTL hits	dbSNPfuncannot
rs10889417	*ALG6*	1	63370230	6			6 altered motifs	8 hits	49 hits	intronic
rs12270446	*GALNTL4*	11	11429816	4	MUS					intronic
rs7128890	*GALNTL4*	11	11407829	4	14 tissues	6 tissues	Pax-5	1 hit		intronic

[1] RegulomeDB (http://www.regulomedb.org)

[2] HaploReg v4.1 (http://archive.broadinstitute.org/mammals/haploreg/haploreg.php)

Abbreviations: SNP, single-nucleotide polymorphism; Chr, chromosome; dbSNPfuncannot, dbSNP function annotation; *ALG6,* ALG6 alpha-1,3-glucosyltransferase; *GALNTL4,* polypeptide N-acetylgalactosaminyltransferase L4.

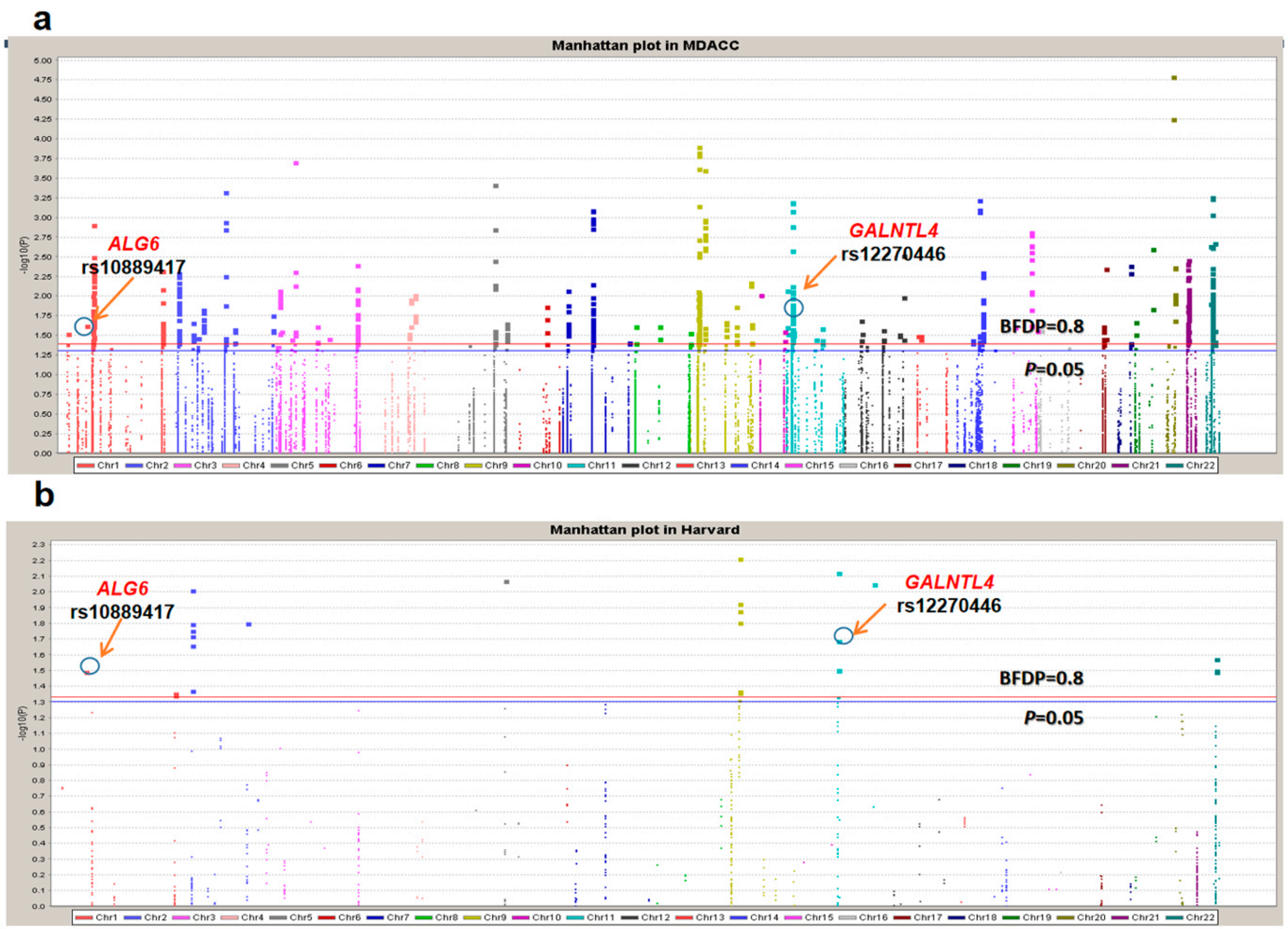

Supplementary Figure S1. Manhattan plots of associations between SNPs and CMSS in the MDACC dataset (**a**) and the NHS/HPFS dataset (**b**). A total of 34,096 (4,770 genotyped and 29,326 imputed) SNPs were included to assess the association between genetic variants and CMSS in the MDACC dataset. The red horizontal line indicates *P* value equal to 0.05 and the blue horizontal line represents BFDP value equal to 0.80. Abbreviations: *ALG6*, ALG6 alpha-1,3-glucosyltransferase; CMSS, cutaneous melanoma-specific survival; *GALNTL4*, polypeptide N-acetylgalactosaminyltransferase L4; MDACC, The University of Texas MD Anderson Cancer Center; NHS/HPFS, the Nurses' Health Study/Health Professionals Follow-up Study; SNP, single nucleotide polymorphism.

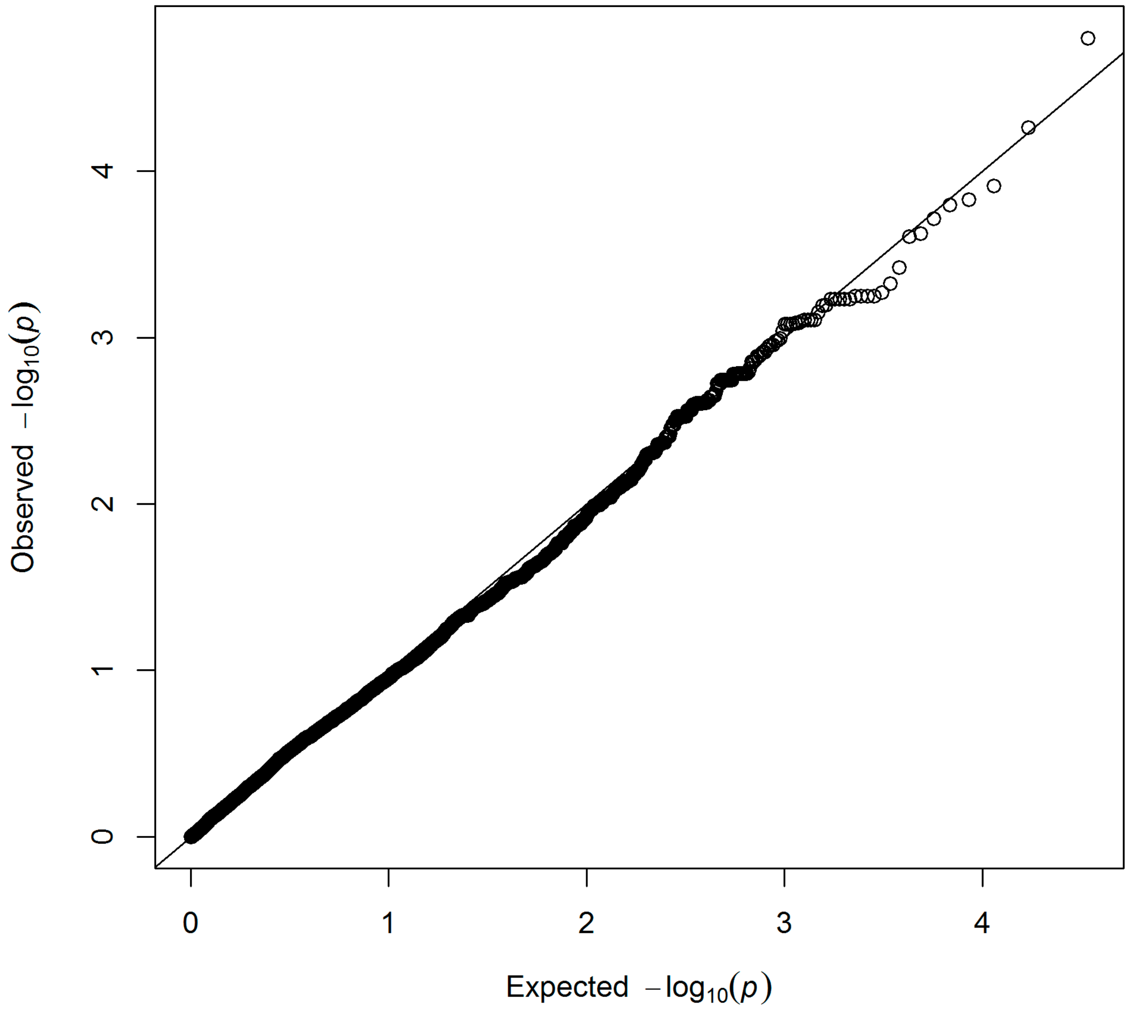

Supplementary Figure S2. Quantile–quantile plot of all SNPs in the MDACC GWAS dataset.

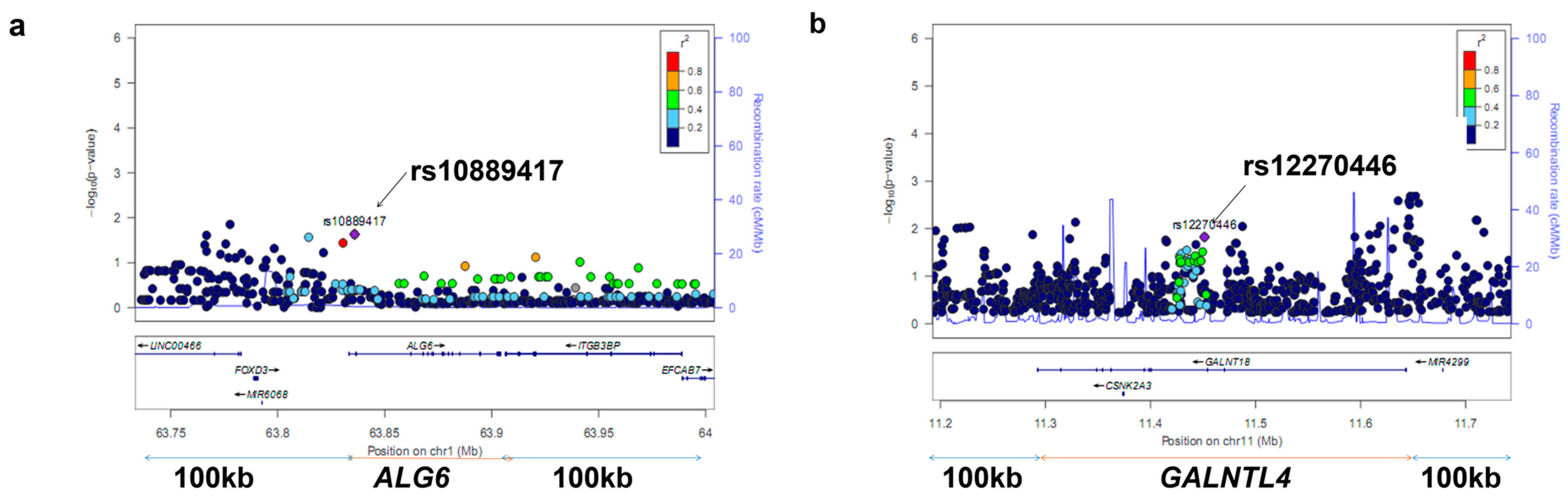

Supplementary Figure S3. Regional association plots contained 100kb up and downstream of the gene regions in (**a**) *ALG6* and (**b**) *GALNTL4*. Abbreviations: *ALG6*, ALG6 alpha-1,3-glucosyltransferase; *GALNTL4*, polypeptide N-acetylgalactosaminyltransferase L4.

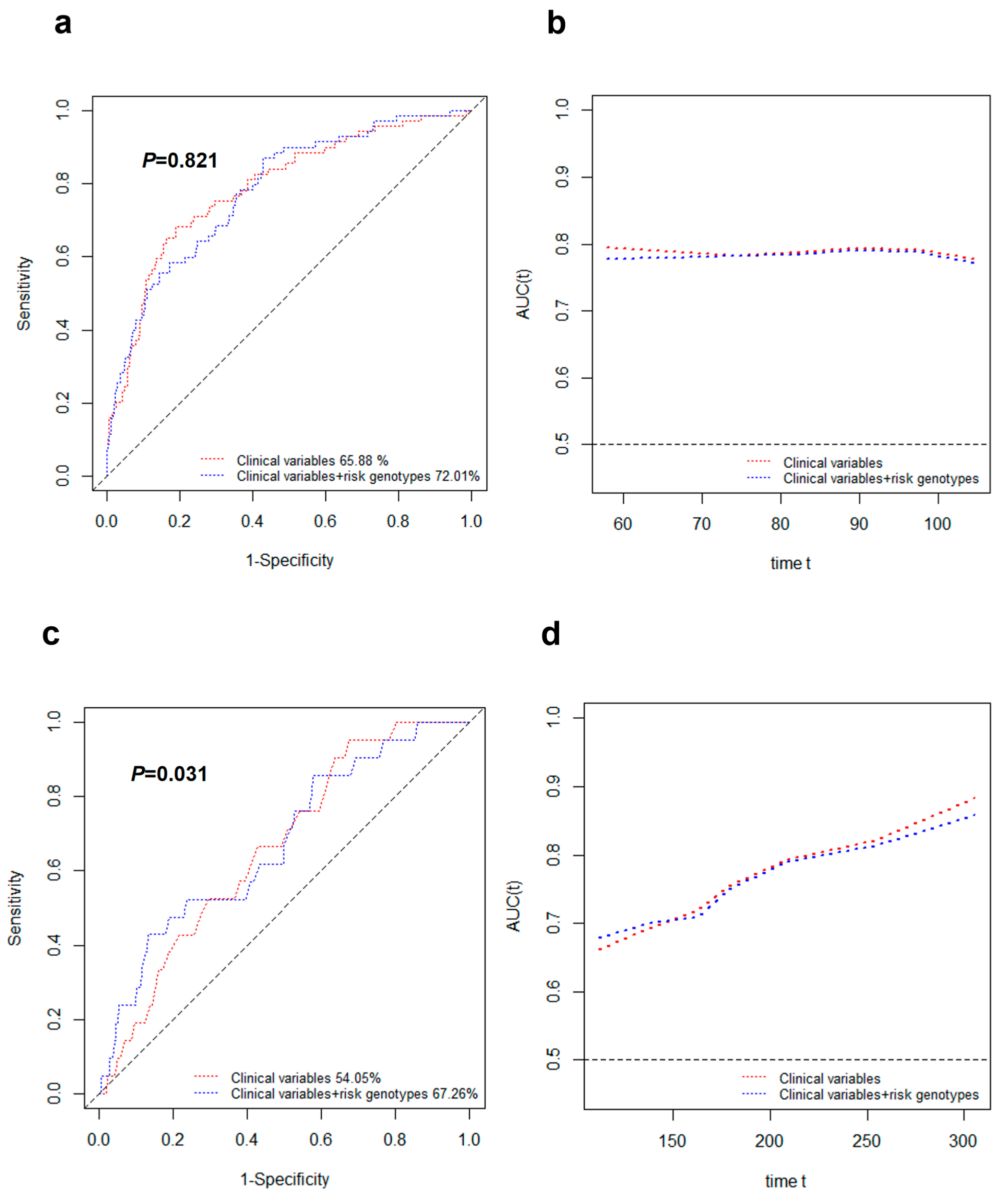

Supplementary Figure S4. Time-dependent AUC and ROC curves for five-year CMSS prediction. ROC curve (**a**) and time-dependent AUC estimation (**b**) for five-year CMSS prediction in CM patients from the MDACC dataset. ROC curve (**c**) and time-dependent AUC estimation (**d**) for five-year CMSS prediction in CM patients NHS/HPFS dataset. Abbreviations: AUC, area under receiver curve; CMSS, cutaneous melanoma-specific survival; MDACC, The University of Texas MD Anderson Cancer Center; NHS, the Nurses' Health Study; HPFS, the Health Professionals Follow-up Study; ROC, receiver operating characteristic.

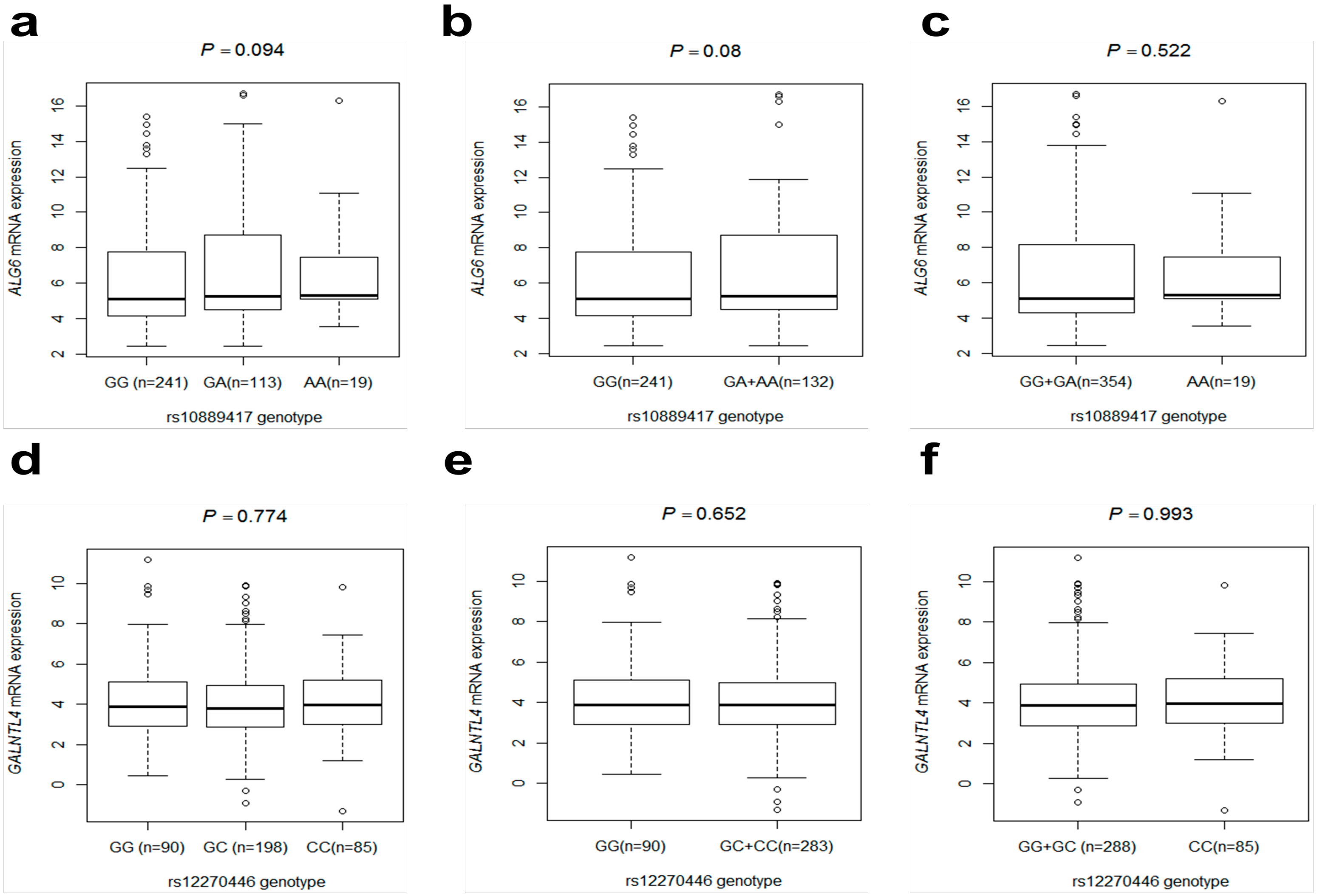

Supplementary Figure S5. Associations between genotypes and their corresponding mRNA expression levels. The eQTL analysis for *ALG6* rs10889417 in 373 European descendants from the 1000 Genomes Project in the (**a**) additive model, (**b**) dominant model and (**c**) recessive model. The eQTL analysis for *GALNTL4* rs12270446 in 373 European descendants from the 1000 Genomes Project in the (**d**) additive model, (**e**) dominant model and (**f**) recessive model. Abbreviations: *ALG6*, ALG6 alpha-1,3-glucosyltransferase; eQTL: expression quantitative trait loci; *GALNTL4*, polypeptide N-acetylgalactosaminyltransferase L4.

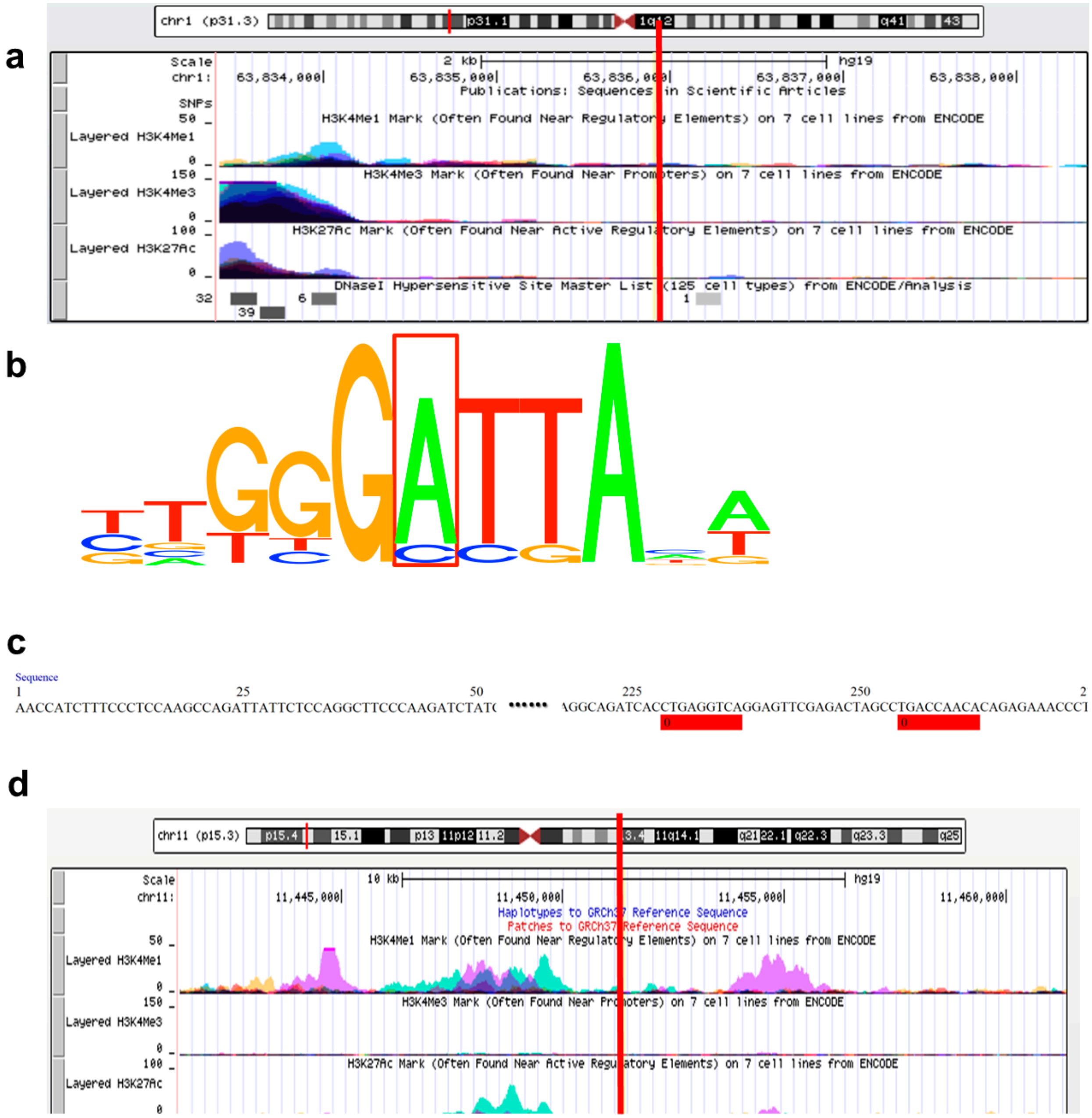

Supplementary Figure S6. Functional prediction of SNPs in the ENCODE project. (a) Location and functional prediction of *ALG6* SNP rs10889417, (b) Position weight matrix based on sequence logo, which showed rs10889417 is located on the Pitx2 motif, (c) The putative Pitx2 binding sites in *ALG6* promoter predicted by PROMO online tools. (d) Location and functional prediction of *GALNTL4* SNP rs12270446.

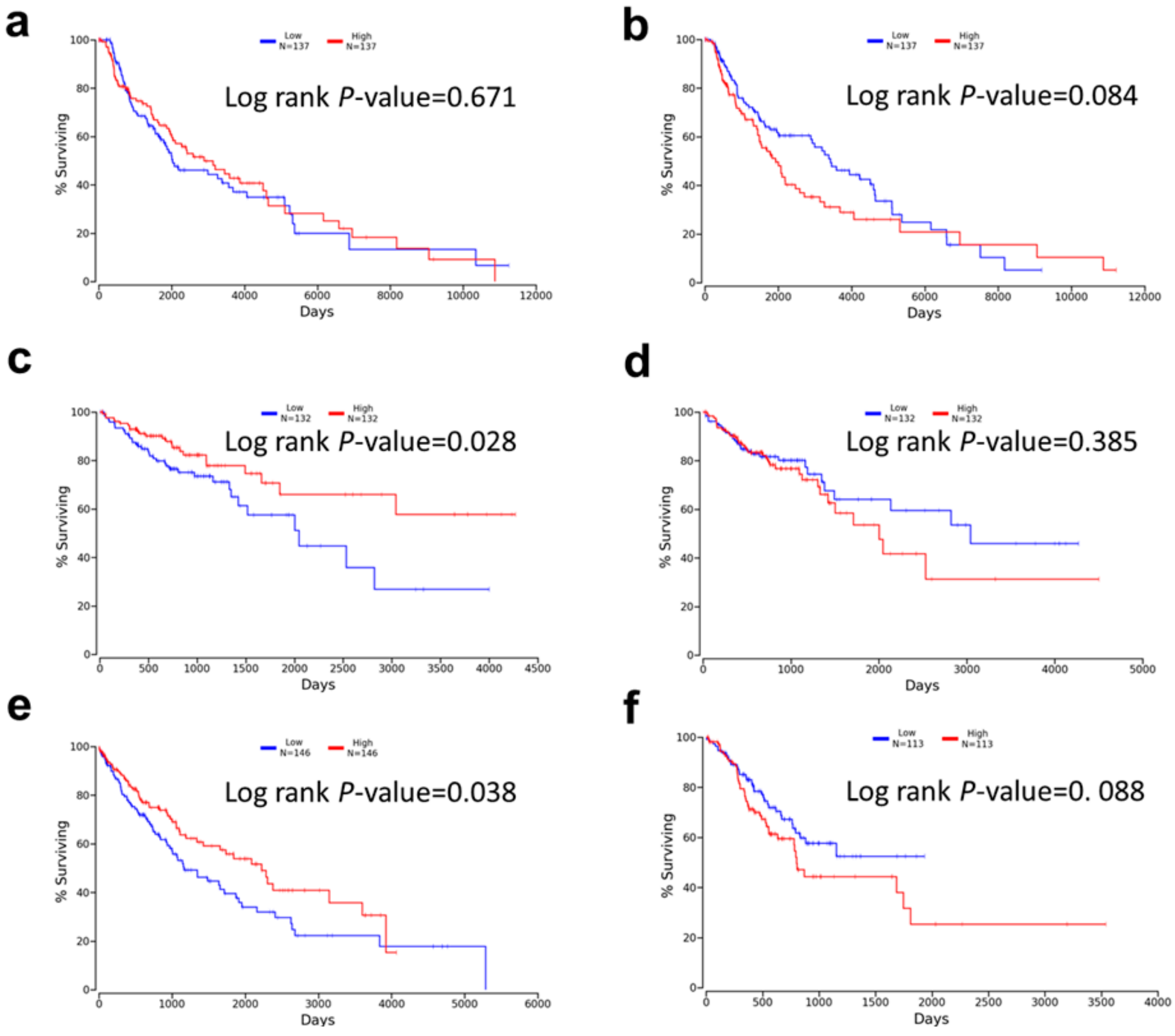

Supplemental Figure S7. Kaplan-Meier analysis for cancer patients by expression levels of *ALG6* and *GALNTL4*: high = above the 30th upper percentile and low = below the 30th lower percentile Based on online survival analysis software (www.oncolnc.org), (a) high *ALG6* expression was associated with a better survival of melanoma patients; (b) low *GALNTL4* expression was associated with a better survival of melanoma patients; (c) high *ALG6* expression was associated with a better survival of colon adenocarcinoma patients; (d) low *GALNTL4* expression was associated with a better survival of colon adenocarcinoma patients; (e) high *ALG6* expression was associated with a better survival of lung squamous cell carcinoma patients; (f) low *GALNTL4* expression was associated with a better survival of stomach adenocarcinoma patients. Abbreviations: *ALG6*, ALG6 alpha-1,3-glucosyltransferase; *GALNTL4*, polypeptide N-acetylgalactosaminyltransferase L4.

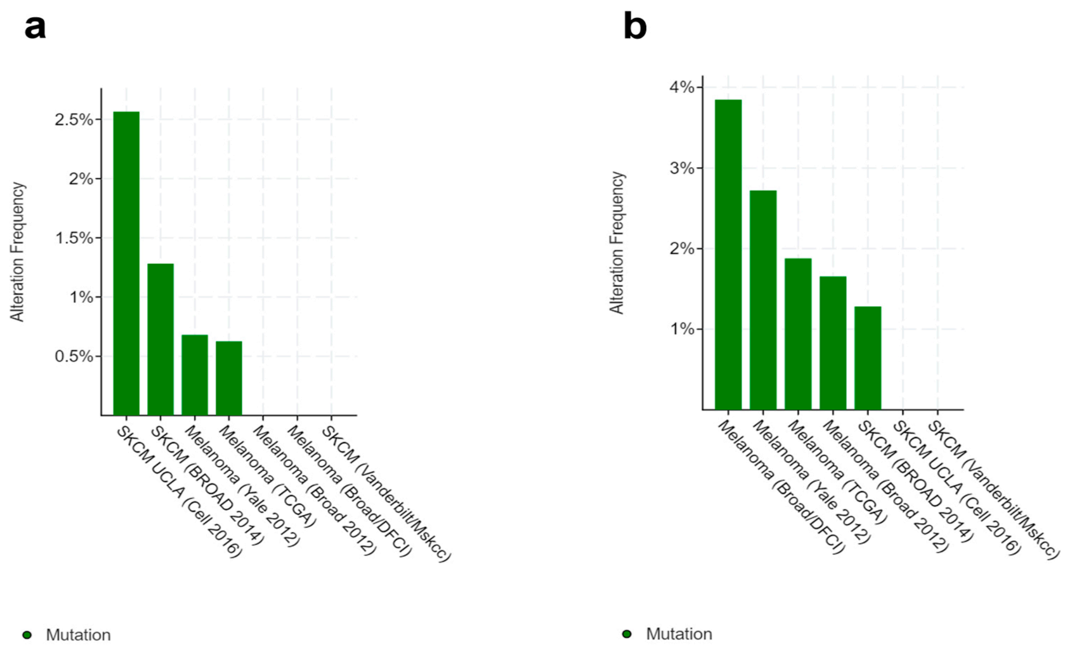

Supplemental Figure S8. Mutation analysis of *ALG6* and *GALNTL4* gene in cutaneous melanoma tumor tissues by using public available data in the database of the cBioportal for Cancer Genomics (http://www.cbioportal.org). (a) *ALG6* had low mutation frequency in melanoma tissues; (b) *GALNTL4* had a relatively high mutation frequency in in melanoma tissues. Abbreviations: *ALG6*, ALG6 alpha-1,3-glucosyltransferase; eQTL: expression quantitative trait loci; *GALNTL4*, polypeptide N-acetylgalactosaminyltransferase.
